# Ability of artificial intelligence to detect T1 esophageal squamous cell carcinoma from endoscopic videos and the effects of real-time assistance

**DOI:** 10.1038/s41598-021-87405-6

**Published:** 2021-04-08

**Authors:** Sho Shiroma, Toshiyuki Yoshio, Yusuke Kato, Yoshimasa Horie, Ken Namikawa, Yoshitaka Tokai, Shoichi Yoshimizu, Natsuko Yoshizawa, Yusuke Horiuchi, Akiyoshi Ishiyama, Toshiaki Hirasawa, Tomohiro Tsuchida, Naoki Akazawa, Junichi Akiyama, Tomohiro Tada, Junko Fujisaki

**Affiliations:** 1 Department of Gastroenterology, Cancer Institute Hospital, Japanese Foundation for Cancer Research, 3-8-31, Ariake, Koto-ku, Tokyo, 135-8550 Japan; 2 Tada Tomohiro Institute of Gastroenterology and Proctology, Saitama, Japan; 3 AI Medical Service Inc., Tokyo, Japan; 4 Division of Gastroenterology and Hepatology, Department of Internal Medicine, Toho University Ohashi Medical Center, Tokyo, Japan; 5 Department of Gastroenterology and Hepatology, Sagamihara Kyodo Hospital, Tokyo, Japan; 6 Department of Gastroenterology and Hepatology, National Center for Global Health and Medicine, Tokyo, Japan; 7 Department of Surgical Oncology, Graduate School of Medicine, The University of Tokyo, Tokyo, Japan

**Keywords:** Cancer screening, Cancer, Gastroenterology

## Abstract

Diagnosis using artificial intelligence (AI) with deep learning could be useful in endoscopic examinations. We investigated the ability of AI to detect superficial esophageal squamous cell carcinoma (ESCC) from esophagogastroduodenoscopy (EGD) videos. We retrospectively collected 8428 EGD images of esophageal cancer to develop a convolutional neural network through deep learning. We evaluated the detection accuracy of the AI diagnosing system compared with that of 18 endoscopists. We used 144 EGD videos for the two validation sets. First, we used 64 EGD observation videos of ESCCs using both white light imaging (WLI) and narrow-band imaging (NBI). We then evaluated the system using 80 EGD videos from 40 patients (20 with superficial ESCC and 20 with non-ESCC). In the first set, the AI system correctly diagnosed 100% ESCCs. In the second set, it correctly detected 85% (17/20) ESCCs. Of these, 75% (15/20) and 55% (11/22) were detected by WLI and NBI, respectively, and the positive predictive value was 36.7%. The endoscopists correctly detected 45% (25–70%) ESCCs. With AI real-time assistance, the sensitivities of the endoscopists were significantly improved without AI assistance (p < 0.05). AI can detect superficial ESCCs from EGD videos with high sensitivity and the sensitivity of the endoscopist was improved with AI real-time support.

## Introduction

Esophageal cancer is the sixth most common cause of mortality worldwide, accounting for almost 508,000 deaths annually^1, 2^. Esophageal squamous cell carcinoma (ESCC) is the most common histological type of esophageal cancer throughout Asia, specifically Japan^3, 4^. ESCC diagnosed in advanced stages often requires invasive treatment and has a poor prognosis; therefore, early detection is important for optimal prognosis^4^. However, early diagnosis remains difficult, and early-stage disease can be overlooked during endoscopic examination.

It can be challenging to correctly diagnose ESCC at early stages using only white light imaging (WLI). Iodine staining can improve ESCC detection with high sensitivity and specificity. However, it can cause severe discomfort and increases the procedure time^5–7^. It is therefore used only for high-risk patients. Narrow-band imaging (NBI) is a revolutionary technology of optical image-enhanced endoscopy that facilitates ESCC detection without iodine staining^8–10^. NBI is easier to use than iodine staining and does not cause patient discomfort. However, NBI has insufficient sensitivity (53%) for detecting ESCC when used by inexperienced endoscopists^11^. Therefore, there is an urgent and unmet need to improve ESCC detection for less experienced practitioners.

Computer-aided diagnosis using artificial intelligence (AI) with deep learning methods could be a useful adjunct to endoscopic examination that could improve detection of early cancers^12–14^. Our group was the first to report good diagnostic performance of AI using deep learning to detect esophageal cancer, including ESCC and adenocarcinoma, from still endoscopic images. In our study, AI had a sensitivity of 98% and could distinguish superficial and advanced cancer with an accuracy of 98%^12^. In superficial cancers, the AI diagnosing system could differentiate pathological mucosal and submucosal microinvasive (SM1) cancers from submucosal deep invasive (SM2) cancers; this can help determine the appropriate treatment course for each patient^15^.

In this study, we evaluated the ability of AI to detect ESCC from esophagogastroduodenoscopy (EGD) videos. Analyzing still images, AI can evaluate only a limited area, however, numerous images are required to screen the entire esophagus, which requires a lot of time. In EGD videos, the whole esophagus can be evaluated without taking pictures of the non-cancerous areas. To ensure that AI would detect ESCC in a fast-moving situation, for example, in the event that an inexperienced endoscopists examines the esophagus too quickly and misses lesions, we prepared two validation video sets which include slow- and high-speed video sets. Analysis of AI diagnosis using videos will aid in realizing the real-time support of AI diagnosing systems for endoscopic examination.

## Methods

This study was approved by the Institutional Review Board of the Cancer Institute Hospital (No. 2016-1171) and the Japan Medical Association (ID JMA-II A00283). Informed consent or an acceptable substitute was obtained from all patients.

### Preparation of training image sets and construction of a convolutional neural network (CNN) algorithm

For this single-center retrospective study, we obtained EGD images taken between February 2016 and April 2017 at the Cancer Institute Hospital, Tokyo, Japan, as described previously^12^. Briefly, we collected 8428 training images of esophageal lesions histologically confirmed to be ESCC or adenocarcinoma. The training esophageal cancer images included 397 ESCC lesions, including of 332 superficial cancers and 65 advanced cancers. Training images included 6026 and 2402 images obtained using WLI and NBI endoscopy, respectively. Poor-quality images resulting from halation, blur, defocus, mucus, and poor air insufflation were excluded. Magnified images obtained by magnifying endoscopy were also excluded. All images of esophageal cancer lesions were manually marked by a well-experienced endoscopist. These images were used to develop a deep learning algorithm using an AI diagnosing system for esophageal cancer.

To develop our AI-based diagnosing system, we used a deep neural network architecture (https://arxiv.org/abs/1512.02325), referred to as a “Single Shot MultiBox Detector”^12^. The Single Shot MultiBox Detector is a deep CNN that consists of 16 layers or more.

### AI system to detection ESCC in videos

**Figure 1:**
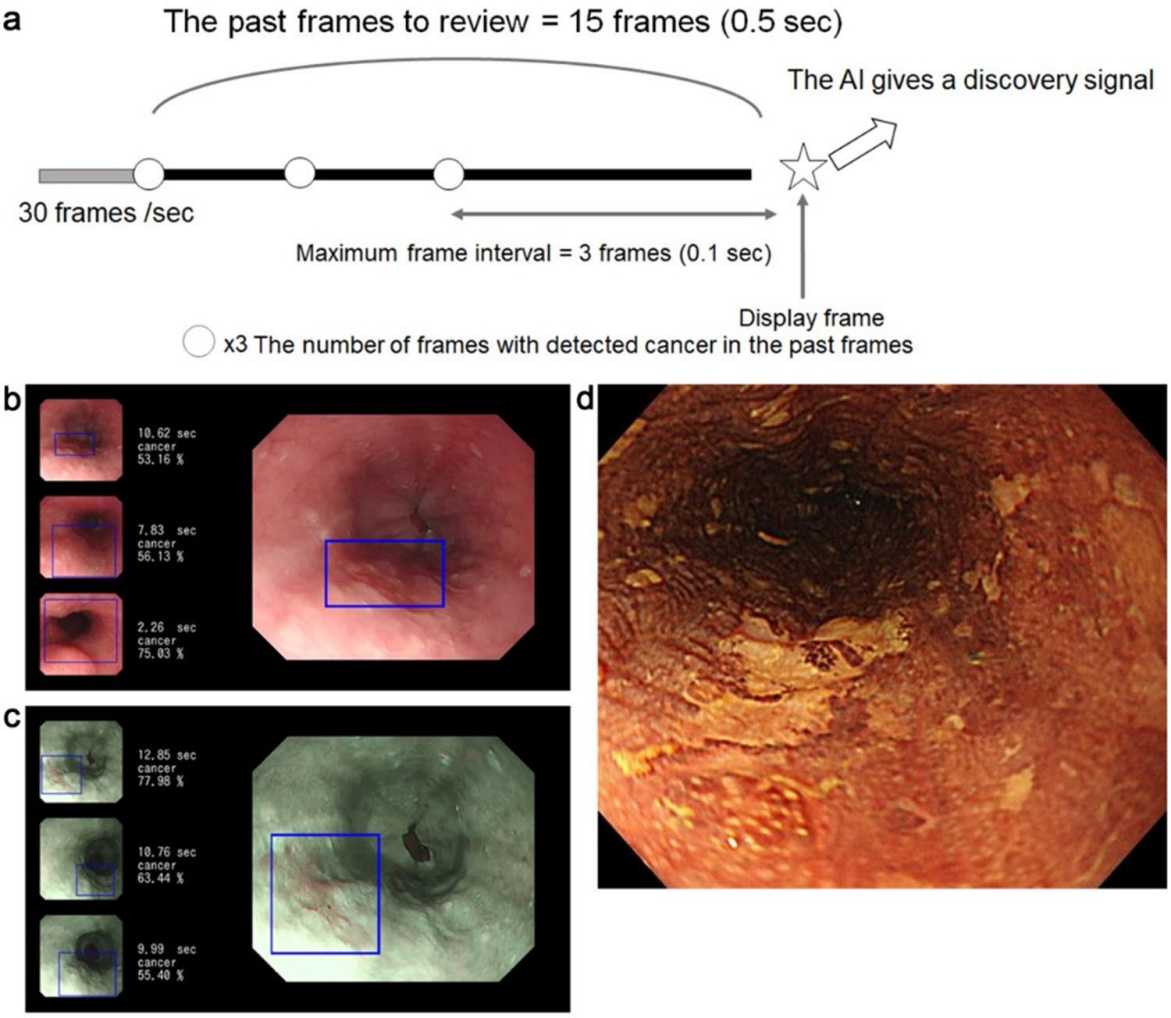
System of AI diagnosis in endoscopic videos and representative images of AI detection of ESCC. (**a**) When the AI detected a cancerous lesion, the AI reviewed the video for 0.5 s (15 frames). If the reviewed section of video included a cancer image in more than 3 frames and the maximum interval from the latest cancer image was 0.1 s (3 frames), the AI diagnosed the lesion as cancer, giving a discovery signal. (**b**,**c**) When the AI recognized a cancerous lesion, a frame was displayed in the endoscopic image surrounding the lesion of interest. The AI inserted the image of the recognized cancer on the left side of the monitor, indicating that it diagnosed the lesion as cancerous. (**d**) When the AI-diagnosed cancer matched the iodine unstained area which was pathologically diagnosed as an ESCC, the AI was considered correct. AI: artificial intelligence, ESCC: esophageal squamous cell carcinoma.

The AI diagnosing system recognized 30 continuous frames of still images in 1 s of video and detected ESCC in the same manner as the analysis of still images. When the AI detected a cancer, it reviewed the video for 0.5 s (15 frames). If the reviewed section included a cancer image in more than 3 frames, and the maximum interval from the latest cancer image was 0.1 s (3 frames), the AI diagnosed the lesion as cancer, giving a discovery signal (Fig. 1a). This setting was based on a small number of videos that were independent of the validation dataset and obtained in a preliminary examination. The AI diagnosing system inserted the image of the recognized cancer on the left side of the monitor (Fig. 1b,c,d), indicating that it diagnosed the lesion as cancerous. If the inserted image included any part of ESCC, we considered it positive, and if the inserted image did not include ESCC, we considered it a false-positive result. Endoscopists could easily verify whether the AI diagnosing system had correctly detected the cancer.

### Validation EGD video set and AI diagnosis

The performance of the AI diagnosing system was evaluated using independent validation EGD videos. We used a total of 144 EGD videos for the two validation sets recorded using high-resolution endoscopes (GIF-H290Z; Olympus Medical Systems, Co, Ltd, Tokyo, Japan). As a slow-speed video validation set, we prepared a dataset of 64 videos of 32 ESCC patients obtained using both WLI and NBI from August 2018 to August 2019 at the Cancer Institute Hospital. In the EGD videos, ESCCs were observed while the endoscope was moving slowly. The whole lesions were observed for 5 to 15 s. When the AI diagnosing system recognized a cancer, the AI indicated it with a bordering square and inserted the image on the left side of the monitor. Because all videos included cancer, we examined only sensitivity in this validation set.

As a high-speed video validation set, we prepared a dataset of 80 videos of WLI and NBI endoscopies performed for 40 patients from August 2018 to August 2019 at the Cancer Institute Hospital. The dataset included 20 patients with 22 superficial ESCC lesions and 20 patients without ESCC. We used EGD videos inserting the endoscope from the cervical esophagus to the esophagogastric junction (EGJ) at a speed of 2 cm/s without stopping or focusing on the specific lesions. These EGD videos were considered to be at the speed at which the endoscopist taking the video passed by without noticing the lesion in routine examination.

Sensitivity, specificity, positive predictive value (PPV), and negative predictive value (NPV) of the AI diagnosing system to detect ESCC from each EGD video were calculated as follows: sensitivity, number of EGD videos in which the AI diagnosing system correctly diagnosed cancer divided by the total number of EGD videos with cancer; specificity, number of EGD videos in which the AI diagnosing system determined that no cancerous lesion existed divided by the total number of videos without cancer; PPV, number of EGD videos in which the AI diagnosing system accurately detected cancer divided by the total number of videos in which the AI diagnosing system detected cancer; and NPV, number of EGD videos in which the AI diagnosing system accurately determined that no cancerous lesion existed divided by the total number of videos that the AI diagnosing system determined as not having a cancerous lesion. In a comprehensive analysis, when the AI diagnosing system detected ESCC in either WLI or NBI videos, we defined this as a correct diagnosis.

### Comparison with endoscopists

We prepared two sets of validation videos for diagnosis by endoscopists. One set was composed of the same set of high-speed videos that the AI diagnosed. The other set was the same as the first set but included the diagnostic real-time assistance of AI, with the AI indicating cancers with a rectangular border without inserting the image on the left side of the monitor. In this video set, we examined the additive effect of the AI system to the diagnostic ability of the endoscopists. These validation video sets were diagnosed by 18 endoscopists, including 7 board-certified endoscopists and 11 non-certified endoscopists, at the Japan Gastroenterological Endoscopy Society. Endoscopists watched high-speed videos on a personal computer and pushed a button when they detected ESCC (correct answer). However, the answer was considered incorrect when an endoscopist failed to push the button and did not recognize the ESCC in the video. Moreover, if endoscopists noticed the lesion but could not confirm that is was an ESCC while the lesion was on the monitor, the answer was considered incorrect. These rules were strictly adhered to, in order to ensure the accuracy of this analysis. The endoscopists could push the button as many times as they detected ESCC. Each endoscopist diagnosed one set of videos chosen randomly. After one month of washout, the endoscopists diagnosed the other validation video set. Between the two rounds of analysis, the endoscopists were not given feedback on their performance or the correct answers.

### Statistical analysis

All continuous variables are expressed as medians and ranges. The differences in AI sensitivity and specificity with WLI and NBI were compared using McNemar's test.

The sensitivities of the endoscopists with or without AI assistance were compared using the Mann–Whitney test with GraphPad Prism software (GraphPad Software, Inc, La Jolla, CA, USA). A p value of < 0.05 was considered statistically significant.

### Human rights statement and informed consent

All procedures followed were in accordance with the ethical standards of the responsible committee on human experimentation (institutional and national) and with the Helsinki Declaration of 1964 and later versions. Informed consent or substitute for it was obtained from all patients for being included in the study.

## Results

### AI diagnosis of the slow-speed validation video set

The characteristics of patients and lesions in the validation video set are summarized in Table 1. In the set, there were more men than women, the median age was 67.5 years, and half of the lesions were located in the middle thoracic esophagus. These characteristics are typical for ESCC in the Japanese population^16^. The median tumor size was 17 mm, and most lesions were mucosal ESCCs (T1a) (Table 1). Therefore, the sensitivity of the AI diagnosing system was 100% for both WLI (32/32) and NBI (32/32).

**Table 1:** Characteristics of the validation video sets (the slow-speed validation video sets).

**Patient characteristics (n = 32)**	
Sex (n), (male/female)	30/2
Age (years), [median(range)]	67.5 (48–84)
**Lesion characteristics (n = 32)**	
Tumor size (mm), [median(range)]	17 (5–52)
Depth of tumor (n), (EP/LPM/MM/SM)	7/21/3/1
Macroscopic type (n), (0-I/0-IIa/0-IIb/0-IIc)	1/2/13/16
Part of esophagus (n)m (Ce/Ut/Mt/Lt)	1/4/16/11
Region (n), (anterior/posterior/left/right)	9/6/7/10

### AI diagnosis of the high-speed validation video set

**Figure 2:**
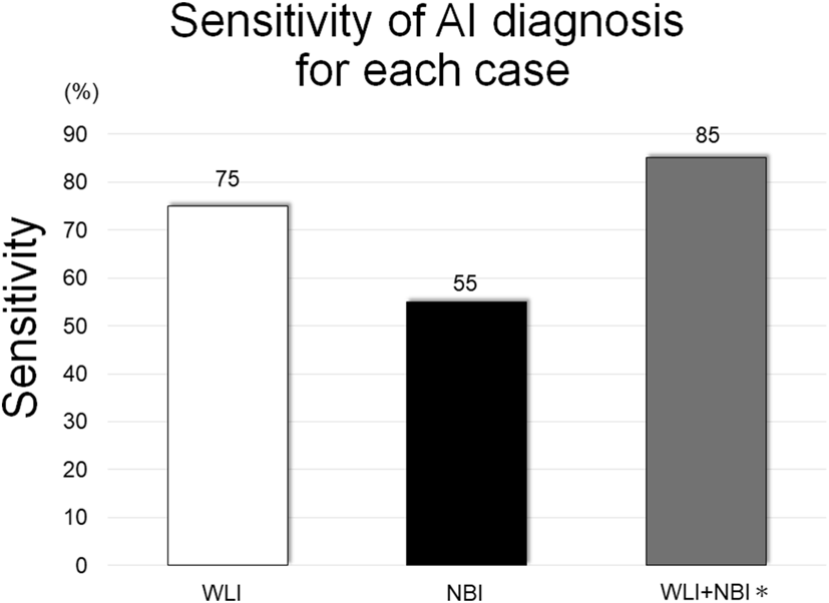
Sensitivity of the AI diagnosis for each case. The sensitivity of the AI diagnosis for each case was slightly higher in WLI than in NBI, but not significantly. * WLI + NBI: when a cancer was diagnosed with either WLI or NBI, we considered that the AI had detected the cancer. AI: artificial intelligence, WLI: white light imaging, NBI: narrow-band imaging.

In the high-speed validation set, 90% patients were men, and the median age was 70 years. The median tumor size was 17 mm with 95% being T1a and 5% being T1b (Table 2). The sensitivity of the AI diagnosing system was 85% (17/20) for the comprehensive diagnosis, whereas the sensitivities based on WLI (Supplementary Video S1) and NBI (Supplementary Video S2) were 75% and 55%, respectively (Fig. 2). The specificity in NBI was significantly higher than that in WLI (80% vs 30%, p < 0.01) (Table 3).

**Table 2:** Characteristics of the validation video sets (the high-speed validation video sets).

**Patient characteristics (n = 40)**	
Sex (n), (male/female)	36/4
Age (years), [median(range)]	70 (57–83)
**Lesion characteristics (n = 22)**	
Tumor size (mm), [median(range)]	17 (5–60)
Depth of tumor (n), (EP/LPM/MM/SM)	8/10/3/1
Macroscopic type (n), (0-I/0-IIa/0-IIb/0-IIc)	0/2/4/16
Part of esophagus (n), (Ce/Ut/Mt/Lt)	0/6/11/5
Region (n), (anterior/posterior/left/right)	8/4/8/2

EP: epithelium, LPM: lamina propria, MM: muscularis mucosae, SM: submucosa Ce: cervical esophagus, Ut: upper thoracic esophagus, Mt: middle thoracic esophagus, Lt: lower thoracic esophagus.

**Table 3:** Detailed results of the AI-based diagnosis for each case.

	Sensitivity	Specificity	PPV	NPV
WLI	75%	30%	52%	55%
	(15/20)	(6/20)	(15/29)	(6/11)
NBI	55%	80%	73%	64%
	(11/20)	(16/20)	(11/15)	(16/25)

PPV: positive predictive value, NPV: negative predictive value, AI: artificial intelligence, WLI: white light imaging, NBI: narrow-band imaging.

### Causes of false-positive and false-negative results in the high-speed video set

**Figure 3:**
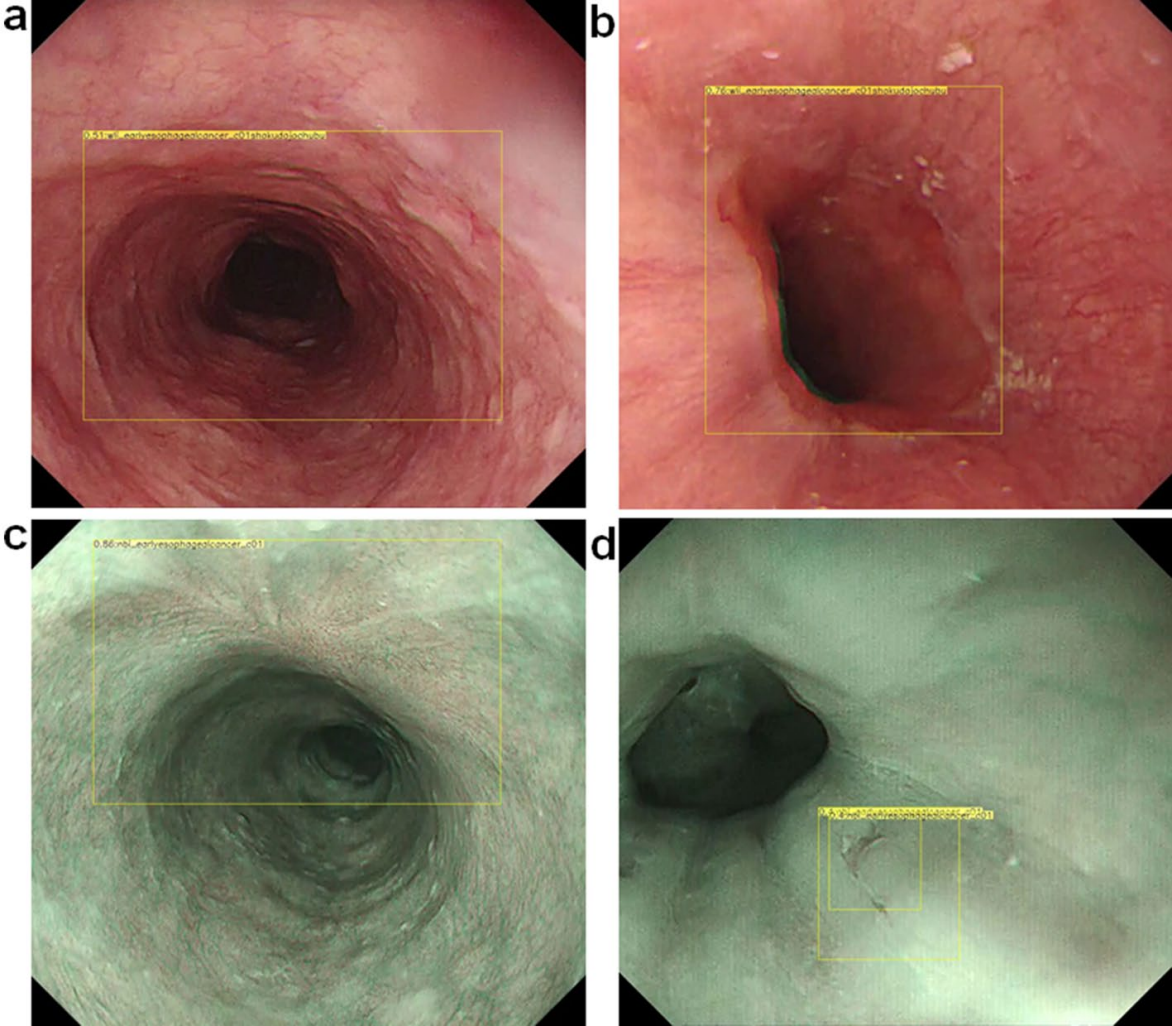
Examples of false-positive images. The yellow squares indicate areas that were misdiagnosed as cancer. (**a**) shadow of lumen, (**b**) EGJ, (**c**) post ER scar, (**d**) inflammation. ER: endoscopic resection, EGJ: esophagogastric junction.

The most frequent cause of false-positive results (Table 4) was a shadow in the esophageal lumen (Fig. 3a), which accounted for 41% of all false-positives. Normal structures and benign lesions, such as the EGJ (Fig. 3b), post-endoscopic resection scars (Fig. 3c), and mucosal inflammation (Fig. 3d) were also misdiagnosed as cancer.

**Table 4:** Details of the false-positive and false-negative lesions.

**False-positive results (n = 74)**	
Shadow of lumen	41% (30)
Inflammation	32% (24)
Post-ER scar	9% (7)
EGJ	18% (13)
**False-negative results (n = 12)**^**a**^	
Inflammation of background mucosa	42% (5)
Anterior wall lesion	33% (4)
Obscure ESCC by WLI	17% (2)
Less than 5 mm in size	8% (1)

ER: endoscopic resection, EGJ: esophagogastric junction, ESCC: esophageal squamous cell carcinoma, WLI: white light imaging.

^a^12 lesions in 11 cases.

**Figure 4:**
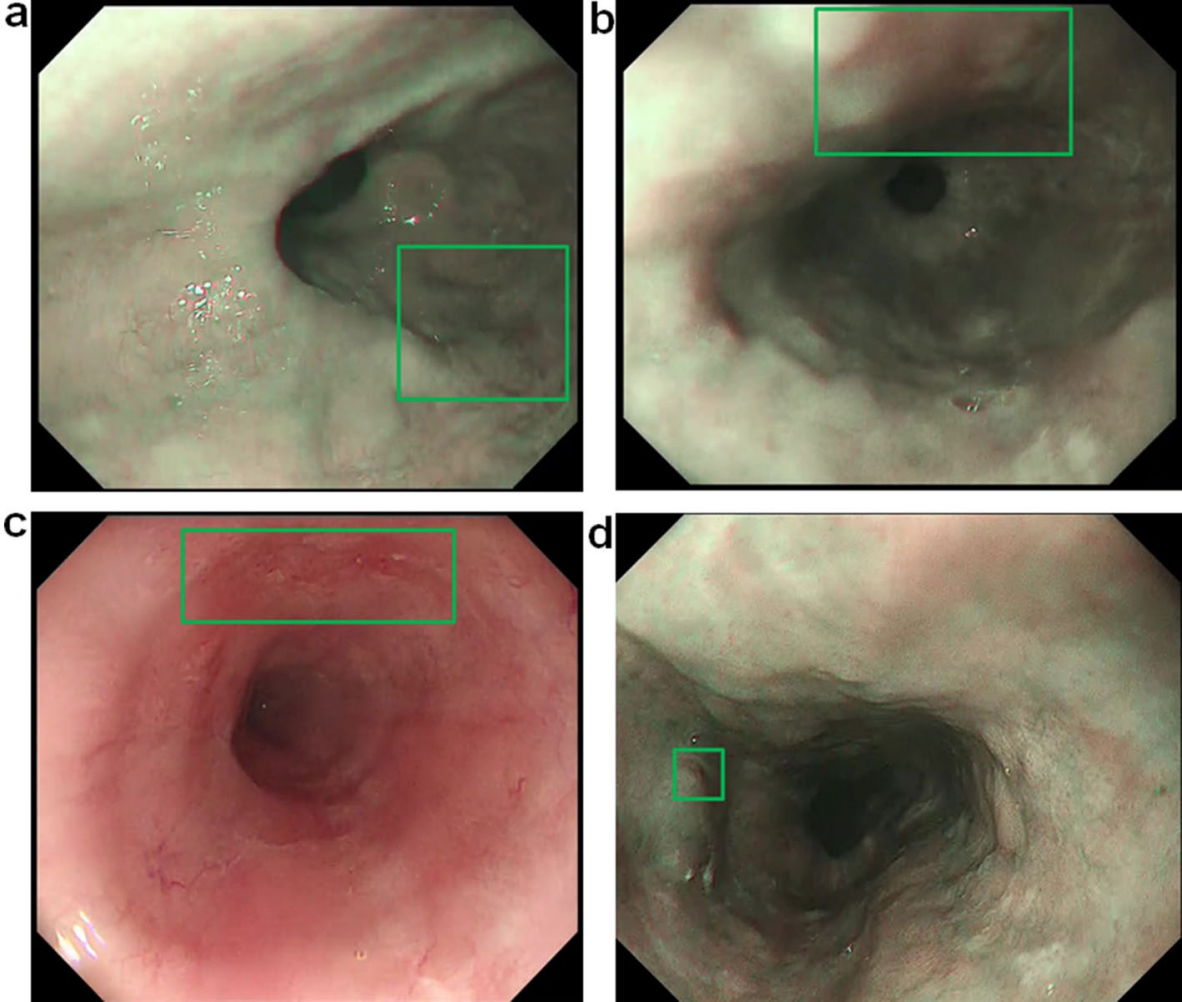
Examples of false-negative images. The cancers in the shown images were missed for the following estimated causes. (**a**) Inflammation of background mucosa, (**b**) anterior wall lesion, (**c**) obscure ESCC by WLI, (**d**) ESCC less than 5 mm. ESCC: esophageal squamous cell carcinoma, WLI: white light imaging.

With regard to false-negative results (Table 4), nearly half the false-negative images were due to esophageal inflammation in the background mucosa (Fig. 4a). Other common causes were anterior wall lesions (Fig. 4b) and obscure ESCC lesions, particularly with WLI endoscopy (Fig. 4c), which were sometimes difficult to be diagnosed even by expert endoscopists. The AI diagnosing system also missed a lesion measuring 5 mm in diameter (Fig. 4d), which was the smallest lesion in the EGD videos. Endoscopists could detect it in the shape of a submucosal tumor-like elevated lesion.

### Time to detection in the high-speed video set

The AI diagnosing system required a median 0.5 s with a range of 0.5–3.63 s to detect a lesion in the validation EGD videos. Although most of the lesions were detected within 1.0 s, the AI diagnosing system required more than 1.0 s to detect 6 lesions. These 6 lesions were larger, with a median size of 30 mm (range, 17–45 mm), with invasion depths of LPM (4 cases) and MM (2 cases); however, 67% were located in the anterior wall and 50% were detected after peristalsis.

### Outcomes of endoscopists in the high-speed validation video set

**Figure 5:**
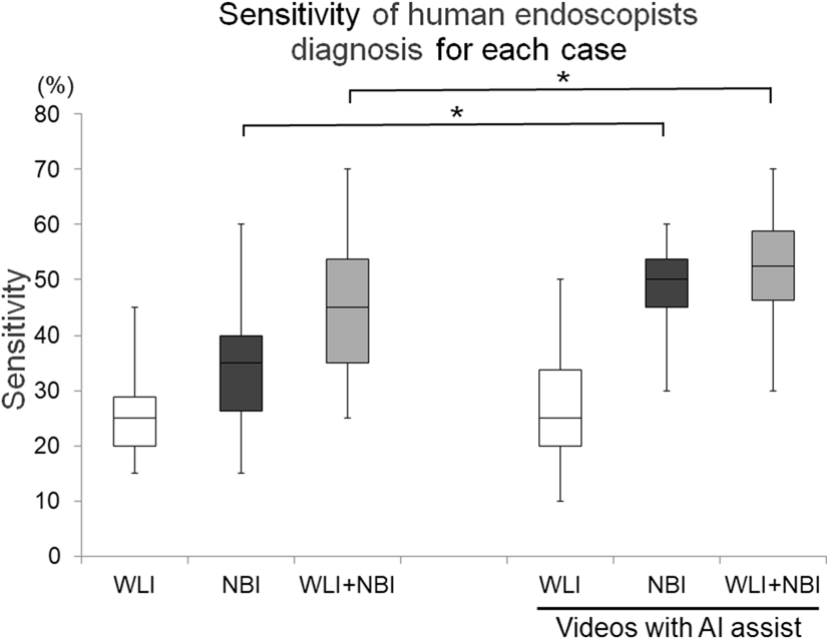
Sensitivity of the endoscopists for each case. The median sensitivity is represented by the center line in the box, which indicates the IQR. The range is indicated by whiskers. When diagnosed with either WLI or NBI, we considered the endoscopists to have detected the cancers (WLI + NBI). IQR: interquartile range, WLI: white light imaging, NBI: narrow-band imaging. *P < 0.05.

The median sensitivity of the endoscopists for the comprehensive cancer diagnosis was 45% (range, 25–70%), whereas the median sensitivities based on WLI and NBI videos were 25% (range, 15–45%) and 35% (range, 15–60%), respectively (Fig. 5). There was no difference in sensitivity between board-certified endoscopists and non-certified endoscopists.

With AI real-time assistance indicating cancers with a rectangle, the sensitivities of endoscopists significantly improved relative to their sensitivities without AI assistance (p < 0.05). The sensitivities improved in 13 of 18 endoscopists by a median of 10% (5–25%). Three of the 5 endoscopists had a decrease of 5–10% in their sensitivity, whilst the remaining 2 remained the same.

## Discussion

We evaluated the computer-aided detection of ESCC from EGD videos that employed AI-based CNN with deep learning. AI diagnosis of ESCCs in videos has been reported recently in other studies^17, 18^. In the videos of these studies, endoscopists carefully observed ESCC lesions and diagnosed them using AI. The videos were similar to the slow-speed video sets in our study, and the results were good. However, it is impossible to observe the whole esophagus carefully for every patient in routine screening examination, as this would require an extended amount of time. As endoscopists are required to detect the lesions first during routine screening examinations, we also tested high-speed video sets. Furthermore, we have shown that the diagnosis of cancers by the endoscopists improved with AI assistance.

We first evaluated slow-speed videos and achieved a detection rate of 100% in both WLI and NBI. We then examined the AI’s performance using high-speed validation videos. The sensitivity of the AI diagnosing system in high-speed videos was 85%, which was much higher than the 45% sensitivity of endoscopists. Their sensitivity significantly improved to 52.5% with AI assistance.

From one point of view, videos are very different from still images. However, AI recognizes 1 s of video as a sequence of 30 frames of still images and detects ESCCs in the same manner as still images of the same quality. In the slow-speed videos, the AI detection rate was 100%, which is consistent with previous reports^17^. Although this is an important step in using AI to diagnose cancer, these results are not sufficient to prove that AI is useful for detecting cancers that humans overlook. To address this in a clinical situation, we used a high-speed validation video set. The sensitivity of the AI diagnosing system was 85% in this high-speed video set. The difference between these two results can be explained by fewer focused clear images in high-speed videos, including more unclear bridging images in between clear images, because the endoscope moved continuously without stopping or focusing on any lesion. It was difficult to detect ESCCs that the scope passed in all consecutive frames in the high-speed videos. It was also more challenging to detect ESCCs during peristalsis, when the ESCC appeared bent or shrunk on the moving esophageal wall, because we only trained the AI system on well-extended esophageal walls. To improve these weaknesses, training videos for an AI system should include plenty of bridging images to achieve higher robustness.

Although the sensitivity of NBI was low and the sensitivity of WLI was slightly high, there was no significant difference between the two-observation method. The number of cases was not large in this study; however, the sensitivity of NBI was sufficiently high with regard to the slow-speed validation set and still image evaluation in previous studies^12^. In addition, it was still higher than the reported PPV of endoscopists examining NBI endoscopies (45% for experienced endoscopists and 35% for less experienced endoscopists)^11^. In daily clinical practice, false-positive results for cancer screening are considered more acceptable than false-negative results. Adding magnifying endoscopy reportedly improves PPV^18, 19^. However, we believe that the AI system without magnifying endoscopy presented here would be most useful for primary detection in clinics or hospitals without well-experienced endoscopists on staff, so we specifically aimed to develop a non-magnifying system in this study.

We also analyzed causes of false-positive and false-negative results. False-positive results were often caused by shadows of the esophageal lumen and EGJ (Table 4), similar to our still image analysis^12^. Nearly half false-negative results were due to inflammation of the background mucosa, which can also be difficult for endoscopists to differentiate. The second most common reason for false-negative results was anterior wall lesions, which can be difficult for endoscopists to detect on tangential views.

The diagnostic speed of the AI system after viewing ESCCs was fast; it could detect the majority of lesions within 1.0 s. However, there were 6 lesions that required more than 1.0 s for detection. It is possible that lesions in the anterior wall and those imaged during peristalsis are difficult to recognize. These 6 lesions were detected after 1.0 s because of their large size; therefore, smaller lesions in the same condition could be missed with fast insertion of the endoscope.

The sensitivity of the AI was better than that of 18 endoscopists using the same videos. The sensitivity of diagnosis by endoscopists was 45%, demonstrating the difficulty in obtaining a proper diagnosis. Moreover, this result suggests that AI could identify 40% ESCCs that were missed by endoscopists. We hypothesize that the low sensitivity of endoscopists was due to the increased speed of the videos and strict criteria of correct answers in which endoscopists had to diagnose the lesions. However, AI could diagnose ESCCs in fast-moving situations that were difficult for endoscopists. Furthermore, we showed the improved diagnostic ability of the endoscopists with AI assistance. Despite AI assistance, endoscopists were less sensitive than AI alone. This is due to the fact that the endoscopist has to make the final decision. Even when ESCC lesions were visible with the help of AI, endoscopists did not make good decisions in a short period of time.

Endoscopists usually move the endoscope quickly through the esophagus, as in the high-speed validation videos, until a suspected cancerous lesion is noticed. They confirm the presence of cancer by observation under magnification or biopsy, although many point out cancer by non-magnifying observation. After detecting the lesion, endoscopists can diagnose it as ESCC by examining a slow-speed video set and still images. AI may help clinicians detect these cancers in real time. After identifying a lesion, the endoscopist should stop to examine it more carefully, as in the slow-speed validation videos. The image of the ESCC that appears on the monitor will let the clinician know that the AI has identified the suspected lesion as cancerous. Diagnostic assistance using the AI system would be helpful in both slow-moving and fast-moving situations.

This study has several limitations. First, this was a single-center, retrospective study. However, we think the results are reliable because the ESCC diagnosis was objectively verified. Second, we moved the endoscope at several speeds to imitate a screening endoscopy; however, we saw an outcome at only two speeds. The AI detected 100% of ESCCs in the slow-speed videos that imitated careful lesion observation. In the high-speed video validation set, the videos imitated endoscope videos without careful lesion inspection. Third, we validated a limited number of ESCCs, but we believe that our previous analysis of still images compensates for the lack of variety in ESCCs in this study. Fourth, we used high-resolution endoscopes, the same used for the training set pictures and videos for the validation set. The sensitivity of AI may be further reduced if poor-resolution endoscopes are used.

## Conclusion

The AI-based diagnostic system demonstrated a high diagnostic accuracy to detect ESCC from recorded EGD videos. Moreover, detection by endoscopists improved with real-time assistance of the AI diagnosing system in high-speed videos. Next, we plan to demonstrate that the AI diagnosing system would be helpful to detect ESCCs in a clinical study. We hope that the AI-based diagnostic system presented here will improve ESCC detection and facilitate earlier diagnosis in daily clinical practice in the near future.

## Supplementary Information

Supplementary Legends.

Supplementary Video S1.

Supplementary Video S2.

## Data Availability

The datasets analysed during the current study are available from the corresponding author on reasonable request.
