# Supplementary material for: Ability of artificial intelligence to detect T1 esophageal squamous cell carcinoma from endoscopic videos and the effects of real-time assistance
Source: Sci Rep. 2021 Apr 8;11:7759. doi: 10.1038/s41598-021-87405-6 (PMC8032773; doi:10.1038/s41598-021-87405-6)
Supplement: Supplementary file 1 — Supplementary Legends. [file 41598_2021_87405_MOESM1_ESM.docx]

**Supplementary Video S1**: AI diagnosed ESCC in WLI in this fast-speed video, in which we inserted the endoscope from the cervical esophagus to the EGJ. Abbreviations - WLI: white light imaging

**Supplementary Video S2**: AI diagnosed ESCC in NBI in this fast-speed video, in which we inserted the endoscope from the cervical esophagus to the EGJ. Abbreviations - NBI: narrow-band imaging
